# Genome-Wide Linkage Study Meta-Analysis of Male Sexual Orientation

**DOI:** 10.1007/s10508-021-02035-3

**Published:** 2021-06-02

**Authors:** Alan R. Sanders, Gary W. Beecham, Shengru Guo, Judith A. Badner, Sven Bocklandt, Brian S. Mustanski, Dean H. Hamer, Eden R. Martin

**Affiliations:** 1 Department of Psychiatry and Behavioral Sciences, NorthShore University HealthSystem, 1001 University Place, Evanston, IL 60201 USA; 2 Department of Psychiatry and Behavioral Neuroscience, University of Chicago, Chicago, IL USA; 3 John P. Hussman Institute for Human Genomics, University of Miami Miller School of Medicine, Miami, FL USA; 4 Dr. John T. Macdonald Foundation Department of Human Genetics, University of Miami Miller School of Medicine, Miami, FL USA; 5 Department of Psychiatry, Rush University, Chicago, IL USA; 6 National Cancer Institute, National Institutes of Health, Bethesda, MD USA; 7 Department of Medical Social Sciences, Northwestern University, Evanston, IL USA

**Keywords:** Chromosome 8, Chromosome Xq28, Complex trait, Genome-wide linkage scan, Sexual orientation

## Abstract

Male sexual orientation is a scientifically and socially important trait shown by family and twin studies to be influenced by environmental and complex genetic factors. Individual genome-wide linkage studies (GWLS) have been conducted, but not jointly analyzed. Two main datasets account for > 90% of the published GWLS concordant sibling pairs on the trait and are jointly analyzed here: MGSOSO (Molecular Genetic Study of Sexual Orientation; 409 concordant sibling pairs in 384 families, Sanders et al. (2015)) and Hamer (155 concordant sibling pairs in 145 families, Mustanski et al. (2005)). We conducted multipoint linkage analyses with Merlin on the datasets separately since they were genotyped differently, integrated genetic marker positions, and combined the resultant LOD (logarithm of the odds) scores at each 1 cM grid position. We continue to find the strongest linkage support at pericentromeric chromosome 8 and chromosome Xq28. We also incorporated the remaining published GWLS dataset (on 55 families) by using meta-analytic approaches on published summary statistics. The meta-analysis has maximized the positional information from GWLS of currently available family resources and can help prioritize findings from genome-wide association studies (GWAS) and other approaches. Although increasing evidence highlights genetic contributions to male sexual orientation, our current understanding of contributory loci is still limited, consistent with the complexity of the trait. Further increasing genetic knowledge about male sexual orientation, especially via large GWAS, should help advance our understanding of the biology of this important trait.

**Supplementary Information:**

The online version contains supplementary material available at 10.1007/s10508-021-02035-3.

## Introduction

Male homosexuality runs in families, and twin studies have shown that genetic contributions appear to account for a moderate proportion of the variation in male sexual orientation with heritability estimated at ~ 32% (for review, see Bailey et al., 2016). Three genome-wide linkage studies (GWLS) have been conducted on male sexual orientation, all focusing on concordant sibling pairs (2010homosexual brothers)—we refer here to these GWLS datasets as Hamer (Mustanski et al., 2005), MGSOSO (Molecular Genetic Study of Sexual Orientation) (Sanders et al., 2015), and Canadian (Ramagopalan et al., ). The Hamer GWLS combined samples from two earlier studies (Hamer et al., 1993; Hu et al., 1995) with newly collected families (Mustanski et al., 2005) to total 155 independent concordant sibling pairs in 145 families. While linkage to chromosome Xq28 was prominent in the earlier linkage studies focusing on chromosome X (Hamer et al., 1993; Hu et al., 1995), the Hamer GWLS instead had its strongest finding of suggestive linkage at chromosome 7q36 (Mustanski et al., 2005). Another research group collected 55 families in Canada and performed a GWLS, with the strongest (albeit not significant) linkage reported at chromosome 14q32 (Ramagopalan et al., 2010). The MGSOSO performed a GWLS on 409 independent concordant sibling pairs in 384 families, making its strongest finding of significant (Lander & Kruglyak, 1995) linkage at pericentromeric chromosome 8 and also detecting suggestive (Lander & Kruglyak, 1995) linkage (supportive evidence of previous findings) at chromosome Xq28 (Sanders et al., 2015). In order to extract the maximal positional information from GWLS of currently available family resources, we jointly analyzed the Hamer and MGSOSO datasets (and included the Canadian dataset by meta-analyzing published summary statistics).

## Method

### Joint Linkage Analyses

The two jointly analyzed datasets used very similar phenotype definitions for homosexual men from their questionnaire data: Hamer used “Kinsey 5–6” for several questions (attraction, fantasy, behavior, and self-identification) (Mustanski et al., 2005), and MGSOSO used “Kinsey 5–6” for fantasy along with homosexual identity (Sanders et al., 2015). The Hamer dataset consisted of 441 individuals in 145 families genotyped with 408 short tandem repeat polymorphism genetic markers (STRPs) (Mustanski et al., 2005), and the MGSOSO dataset consisted of 908 individuals in 384 families and genotyped with 45,387 single-nucleotide polymorphism genetic markers (SNPs) (Sanders et al., 2015). Various quality control steps had already been performed in the respective GWLS as previously detailed (Mustanski et al., 2005; Sanders et al., 2015). After obtaining collaborative access to genotypes for each dataset, we conducted multipoint nonparametric linkage analyses with Merlin v1.1.2 (Abecasis et al., 2002) on the Hamer (Mustanski et al., 2005) and MGSOSO (Sanders et al., 2015) datasets separately since they were genotyped differently (STRPs vs. SNPs). To integrate, we found the genetic positions of the respective markers in the Rutgers Map v.3 (hg19 build) (Nato et al., 2018) and then used the nonparametric S-pairs and grid 1 cM options to perform multipoint linkage on both data sets, followed by combining LOD scores at each grid position across the marker sets.

### Meta-Analyses of Summary Statistics

**Fig. 1:**
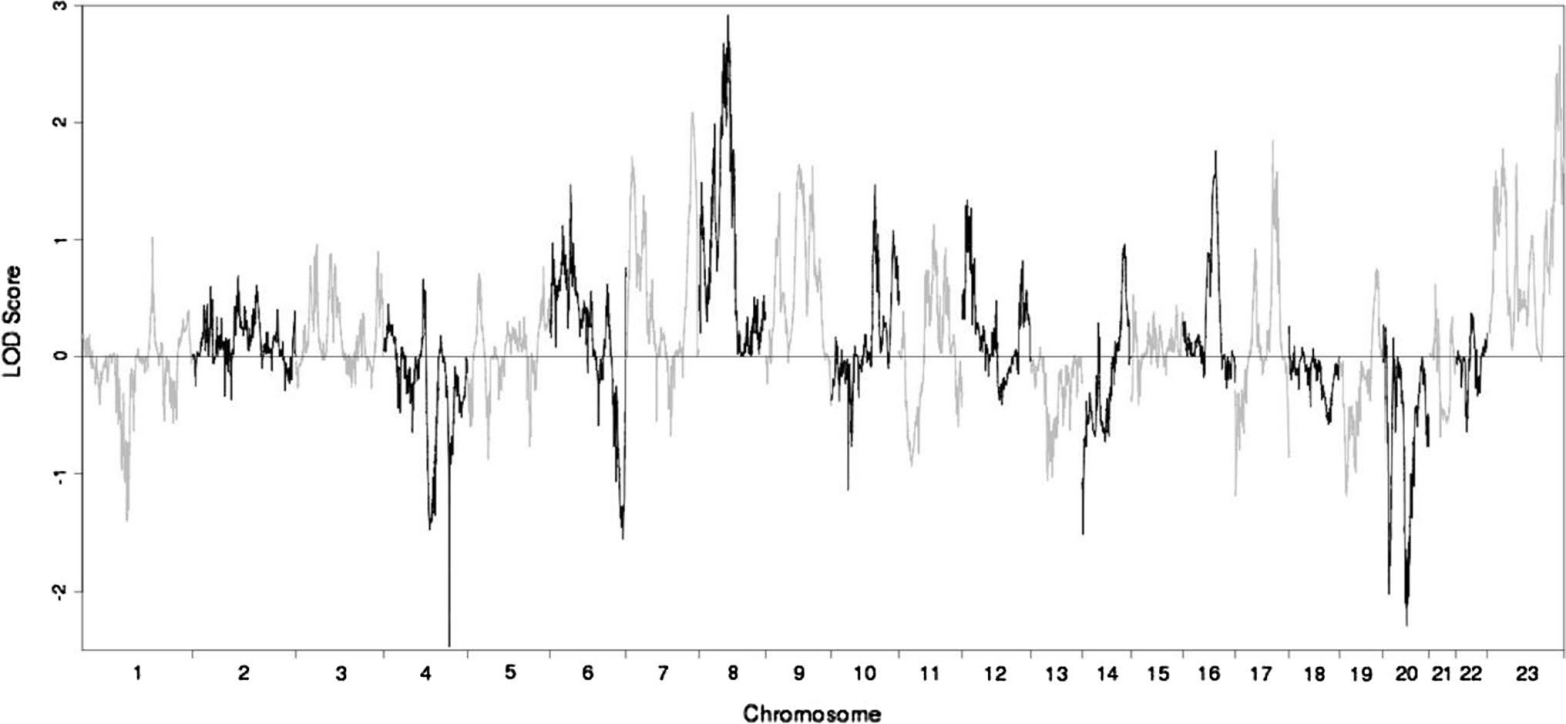
Joint multipoint linkage analysis of the combined Hamer and MGSOSO datasets. Multipoint LOD scores are plotted v. the chromosomal positions for the nonparametric linkage analysis. Adjacent chromosomes are separated by alternating black and gray lines

For phenotype definitions for homosexual men, the Canadian dataset used an interview approach based on identity and corroboration by sibling, and on a sub-sample all also had Kinsey 5–6 for several questions (attraction, fantasy, and behavior) (Rice et al., 1999a, b). As we were unable to access genotypes for the Canadian dataset (accounting for < 10% of the families in GWLS on the trait), we were only able to incorporate the Canadian GWLS by meta-analyzing summary statistics. Thus, we used the plotted multipoint Canadian GWLS Fig. 1 (Ramagopalan et al., 2010) and interpolated into cM bins enabling use of GWLS meta-analytic methods not needing genotypes, namely the multi-scan probability (MSP) approach utilizing regional *p*-values (Badner & Gershon, 2002), and the rank-based genome scan meta-analysis (GSMA) approach (Levinson et al., 2003; Wise & Lewis, 1999).

## Results

**Fig. 2:**
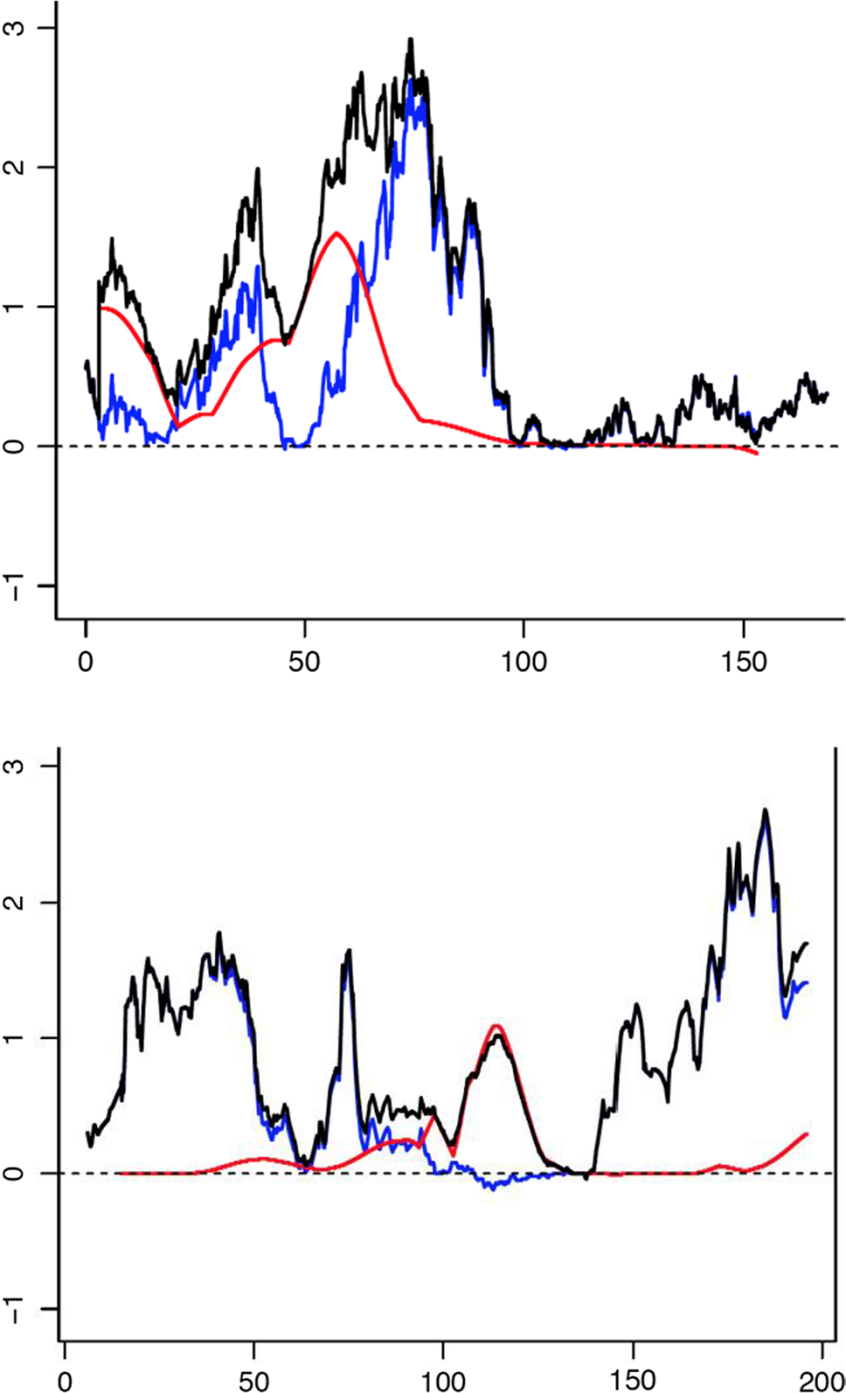
Individual chromosomal plots of multipoint linkage peaks on chromosomes 8 and X. Multipoint LOD scores are plotted v. the chromosomal positions for the nonparametric linkage analysis, the red line indicating the results from the Hamer dataset, the blue line for the MGSOSO dataset, and the black line for the joint analysis. Chromosome 8 (top) has its multipoint linkage peak in the pericentromeric region, and chromosome X (bottom) has its multipoint linkage peak at Xq28

The multipoint plots for the Hamer and the MGSOSO datasets for the current analyses (Supplementary Figs. 1 and 2, respectively) line up very well with the original GWLS manuscripts’ multipoint plots–Fig. 1a (Mustanski et al., 2005) and Fig. 1 (Sanders et al., 2015), respectively. This overlap of multipoint findings was found despite some differences between the original reports (Mustanski et al., 2005; Sanders et al., 2015) and the current manuscript in statistical analysis software (Aspex vs. Merlin for the Hamer dataset) and genetic map used (deCode vs. Rutgers for both the Hamer and MGSOSO datasets). The joint analysis of the combined Hamer and MGSOSO datasets is shown in Fig. 1, with zoomed-in plots of the top two multipoint linkage peaks from this joint GWLS depicted for chromosomes 8 and X in Fig. 2. The results of the meta-analyses of summary statistics from Hamer, MGSOSO, and Canadian GWLS datasets are presented in Supplementary Tables 1 (MSP) and 2 (GSMA).

## Discussion

Our primary analysis for this investigation was the joint analysis of multipoint linkage from the Hamer and MGSOSO datasets (Mustanski et al., 2005; Sanders et al., 2015), to which each dataset contributed some peaks (Fig. 1, Supplementary Figs. 1 and 2). Overall, the maximum multipoint peaks increased little in height, though the pericentromeric chromosome 8 peak was broadened (Fig. 2). Chromosomes 8 and X retained the highest multipoint peaks genome-wide, mostly arising from the larger (MGSOSO) dataset (Fig. 2). The joint analysis gives a more comprehensive picture of shared and heterogeneous linkage regions (e.g., at pericentromeric chromosome 8), the studies share overlapping peaks (possibly suggesting heterogeneity, perhaps with different genes involved in the different datasets), and the evidence broadens the search. The secondary analyses on summary statistics using MSP and GSMA to incorporate all three (Hamer, MGSOSO, Canadian) GWLS datasets showed no genome-wide significant results though suggestive findings remained present. The joint analysis of multipoint linkage (Fig. 1) extracted the available positional information from collaborating GWLS, though previous GWLS findings were not much further strengthened in these analyses. Nevertheless, this provides information to complement other approaches, such as helping prioritize findings from GWAS. Linkage and association studies measure different genetic properties (i.e., segregation of a region within families, vs. correlation of alleles in a population), both of which provide clues about underlying trait genetics. Thus, since GWLS are different from GWAS, we were unable to directly combine any GWAS (e.g., Ganna et al., 2019) with the studied GWLS in our GWLS meta-analysis. Limitations include those inherent to linkage (as opposed to GWAS) of traits with complex genetics (e.g., their limited utility for phenotypes with contributions from more than one or a few genes); on the other hand, linkage retains some advantages over association approaches, such as being robust to allelic heterogeneity (Lipner & Greenberg, 2018). Accumulating genetic studies of the trait such as by much enlarged GWAS (e.g., Ganna et al., 2019) will be especially useful, given its successful application in the study of other phenotypes manifesting complex genetics (e.g., Fig. 3b in Sullivan et al. (2018)).

## Supplementary Information

Below is the link to the electronic supplementary material.

Supplementary file1 (DOCX 27552 kb)
