# Supplementary material for: Genome-Wide Linkage Study Meta-Analysis of Male Sexual Orientation
Source: Arch Sex Behav. 2021 Jun 2;50(8):3371–5. doi: 10.1007/s10508-021-02035-3 (PMC8604844; doi:10.1007/s10508-021-02035-3)
Supplement: Supplementary file 1 — Supplementary file1 (DOCX 27552 kb) [file 10508_2021_2035_MOESM1_ESM.docx]

**Supplementary Figure 1. Multipoint LOD scores for the Hamer dataset.**

**Supplementary Figure 2. Multipoint LOD scores for the MGSOSO dataset.**

**Supplementary Figure 1. Multipoint LOD scores for the Hamer dataset.**


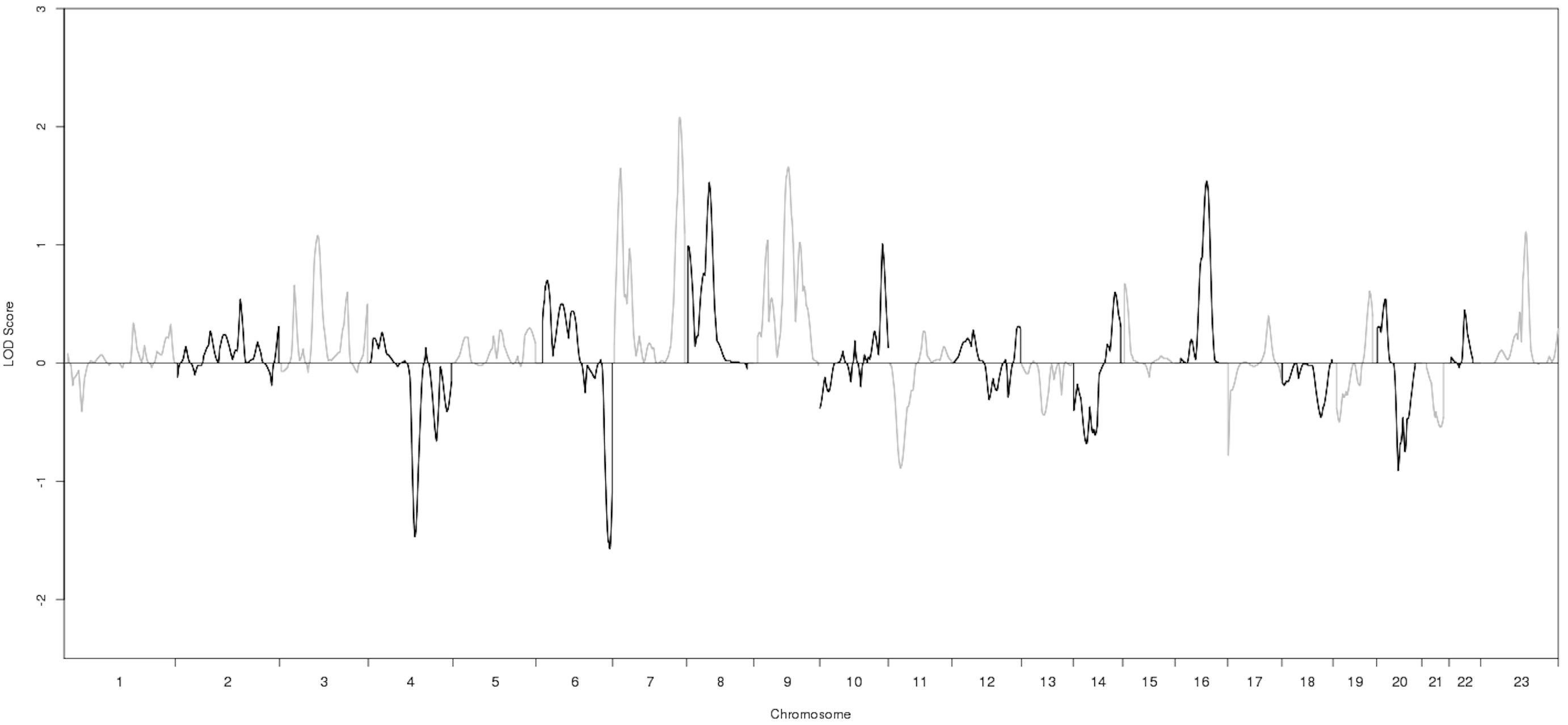


**Supplementary Figure 2. Multipoint LOD scores for the MGSOSO dataset.**


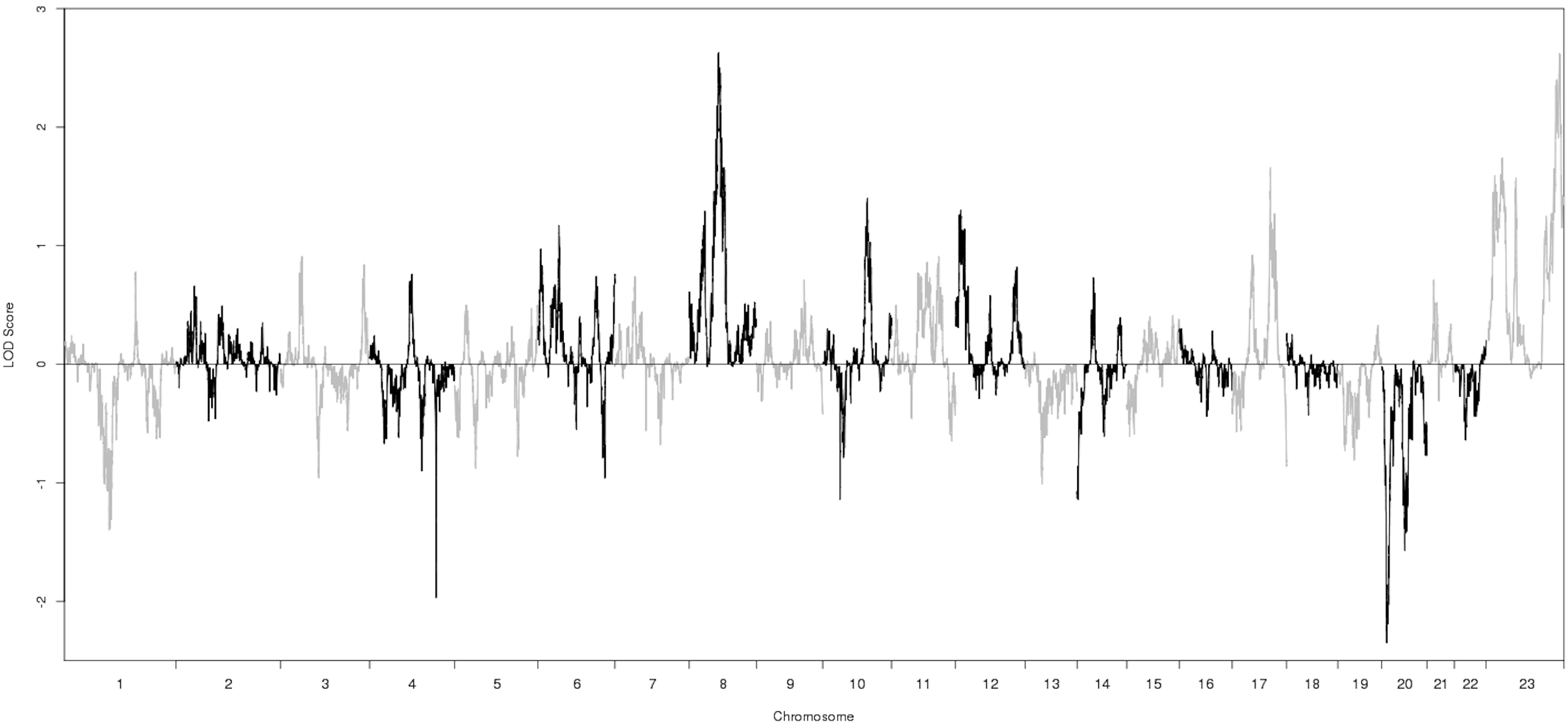


**Supplementary Table 1. Top results from the Multi-Scan Probability (MSP) approach.**

| **Chr** | **cM** | **Multipoint LOD Scores** | | | **Multipoint *p*-values** | | | **Combination** | |
| --- | --- | --- | --- | --- | --- | --- | --- | --- | --- |
|  |  | **MGSOSO** | **Hamer** | **Canadian** | **MGSOSO** | **Hamer** | **Canadian** | **MSP_all** | **LOD equiv** |
| 14 | 98.8 | -0.03 | 0.36 | 2.80 | 0.64 | 0.099 | 0.00016 | 0.00082 | 2.15 |
| 8 | 74.0 | 2.62 | 0.30 | -1.00 | 0.00026 | 0.12 | 0.98 | 0.0020 | 1.80 |
| X | 185.0 | 2.61 | 0.05 | 0.00 | 0.00026 | 0.32 | 0.50 | 0.0026 | 1.70 |
| 9 | 120.3 | 0.71 | 0.92 | 0.27 | 0.035 | 0.020 | 0.13 | 0.0049 | 1.45 |
| X | 40.8 | 1.74 | 0.04 | 0.20 | 0.0023 | 0.33 | 0.17 | 0.0065 | 1.34 |
| 7 | 169.7 | 0.01 | 2.08 | 0.00 | 0.42 | 0.00098 | 0.50 | 0.0093 | 1.20 |
| 17 | 96.6 | 1.66 | 0.19 | -0.10 | 0.0028 | 0.17 | 0.75 | 0.015 | 1.02 |
| 16 | 83.4 | 0.28 | 1.48 | -0.20 | 0.13 | 0.0045 | 0.83 | 0.018 | 0.95 |
| 6 | 53.3 | 1.17 | 0.30 | 0.00 | 0.010 | 0.12 | 0.50 | 0.022 | 0.88 |
| 7 | 15.7 | 0.26 | 1.43 | -0.80 | 0.14 | 0.0051 | 0.97 | 0.024 | 0.85 |
| 14 | 16.5 | -0.24 | -0.27 | 1.60 | 0.85 | 0.87 | 0.0033 | 0.062 | 0.52 |
| 19 | 65.9 | 0.00 | -0.19 | 1.20 | 0.50 | 0.83 | 0.0094 | 0.085 | 0.41 |

^a^ Abbreviations include: Chr (chromosome), cM (centimorgans), MSP_all (multi-scan probability for the combined datasets), and LOD equiv (LOD score equivalent for the corresponding MSP_all).

**Supplementary Table 2. Top results from the Genome Scan Meta-Analysis (GSMA) approach.**

| **Unweighted** | | | |  | **Weighted by sample size** | | | |
| --- | --- | --- | --- | --- | --- | --- | --- | --- |
| **Chr** | **Bin** | **SR*p*value** | **ORpvalue** |  | **Chr** | **Bin** | **SR*p*value** | **ORpvalue** |
| 6 | 0-30 | 0.017 | 0.92 |  | X | 60-90 | 0.0098 | 0.76 |
| 7 | 30-60 | 0.018 | 0.72 |  | X | 30-60 | 0.015 | 0.65 |
| X | 60-90 | 0.021 | 0.56 |  | 6 | 0-30 | 0.017 | 0.42 |
| 8 | 0-30 | 0.022 | 0.35 |  | 17 | 90-120 | 0.024 | 0.40 |
| 9 | 90-120 | 0.023 | 0.19 |  | X | 180-210 | 0.029 | 0.37 |
| 1 | 150-180 | 0.031 | 0.24 |  | 12 | 0-30 | 0.031 | 0.24 |
| 14 | 90-120 | 0.031 | 0.11 |  | 8 | 0-30 | 0.035 | 0.18 |
| 12 | 150-180 | 0.038 | 0.12 |  | 7 | 30-60 | 0.036 | 0.092 |
| X | 30-60 | 0.044 | 0.12 |  | 1 | 150-180 | 0.038 | 0.047 |
| 17 | 90-120 | 0.050 | 0.12 |  | 12 | 150-180 | 0.039 | 0.022 |

^a^ Abbreviations include: Chr (chromosome), BIN (cM boundaries of bin), SR*p*value (*p*-value for the Summed Rank analysis, based on simulating the by-bin LOD scores within a study), and OR*p*value (*p*-value for the Ordered Rank analysis, based on number of nth-position SRs that exceed the observed SR). Both *p*-values are nominal values. Genome-significance would correct for the 137 bins tested (i.e., *p* < 0.05 / 137 = 0.00036).

**Supplementary Text.**

**Potential Sample Overlap.** Aspects of sample recruitment are described elsewhere for the Hamer (Hamer, 1999; Hamer et al., 1993; Hu et al., 1995; Mustanski et al., 2005), Canadian (Ramagopalan et al., 2010; Rice et al., 1999a, 1999b), and MGSOSO (Sanders et al., 2015) samples. Briefly, for recruitment there was little overlap for geography (especially for Canadian sample with others) or timing (of MGSOSO versus others), but more overlap of methods (all used some degree of advertising in LGBT media). It is noteworthy that the largest study, MGSOSO, specifically asked whether potential participants had participated in any previous genetic study and excluded any such families from analyses. This makes it unlikely that there was substantial (or likely any) subject overlap among the three samples. However, despite the claim and assumption of fully independent samples, such a possibility of family overlap remains and is not amenable to direct examination due to genotype unavailability (Canadian sample) and due to genotyping differences (Hamer sample with STPRs, MGSOSO sample with SNPs).

**Relationship Confirmation.** The Canadian GWLS (Ramagopalan et al., 2010) reported using Pedstats (Wigginton & Abecasis, 2005) to verify pedigrees (i.e., confirm self-reported relationships) already. The Hamer GWLS (Mustanski et al., 2005) did not report any attempt to confirm self-reported relationships, however, by using PREST (McPeek & Sun, 2000), we confirmed self-reported relationships, including that all analyzed sibling pairs were full-sibling pairs. The MGSOSO GWLS (Sanders et al., 2015) already checked pedigree structure using GRR version 1.2.1.41 (Abecasis, Cherny, Cookson, & Cardon, 2001), which detected some half-sibling pairs that were then correctly specified in the pedigree files prior to analyses as previously reported (Sanders et al., 2015).

**Information Content.** While the information content (IC) of the genetic markers was likely high with the Canadian GWLS (Ramagopalan et al., 2010) since it used a dense SNP panel (~6,000 SNPs from the Illumina HumanLinkage-12 BeadChip Infinium array), the IC details (and genotypes) were unavailable. The MGSOSO GWLS (Sanders et al., 2015) used an even denser set of SNPs, ~45,000 SNPs (after linkage disequilibrium [LD] pruning) from the Affymetrix 5.0 Genotyping Array, and had high IC (mean 0.79). The IC (mean 0.67) was lower, as expected, for the Hamer GWLS (Mustanski et al., 2005) since it used ~400 STRP markers from the ABI PRISM Linkage Mapping Set Version 2.5. This was consistent for chromosomes 8 and X, where average ICs for the STRP mapping panel used by Hamer (chromosome 8 average IC = 0.63 and chromosome X average IC = 0.81) were lower than the much denser SNP coverage available for the MGSOSO GWLS (chromosome 8 average IC = 0.79 and chromosome X average IC = 0.94).

**Minimizing Type 1 error due to Intermarker Linkage Disequilibrium (LD).** Using markers in high LD with each other could contribute to false positive findings in multipoint linkage analyses, especially in the absence of genotyped parents or additional siblings (Huang, Shete, & Amos, 2004). To minimize this problem, two of the GWLS included as many genotyped parents and heterosexual siblings as available: The Hamer GWLS (Mustanski et al., 2005) included 90 genotyped parents in their 146 studied families (note that we had 145 of these 146 families available for the meta-analysis), and 46 of these families had one or more genotyped heterosexual siblings. The MGSOSO GWLS (Sanders et al., 2015) included 82 genotyped parents and 33 genotyped heterosexual siblings in its 384 studied families. The Canadian GWLS (Ramagopalan et al., 2010) only genotyped homosexual brothers–no parents or heterosexual siblings were genotyped. Additionally, the three GWLS avoided markers in high LD with each other by the nature of the utilized marker set or by pruning: The Canadian GWLS (Ramagopalan et al., 2010) used a commercial array (Illumina HumanLinkage-12 BeadChip Infinium array) with ~6,000 SNPs that were in low LD with each other. The Hamer GWLS (Mustanski et al., 2005) used a commercial set (ABI PRISM Linkage Mapping Set Version 2.5) with ~400 STRPs dispersed across the genome (average of 10 cM intermarker distance). The MGSOSO GWLS (Sanders et al., 2015) used a commercial array (Affymetrix 5.0 Genotyping Array), but used PLINK to remove (prune) those in high LD with each other, resulting in ~45,000 SNPs with low intermarker LD (i.e., removed those with r^2^ > 0.16, a cutoff and measure based on previous analyses (Boyles et al., 2005)). These efforts minimized Type 1 error for multipoint linkage analyses due to intermarker LD, as suggested elsewhere (Huang et al., 2004).
